# Dynamic immune status analysis of peripheral blood mononuclear cells in patients with *Klebsiella pneumoniae* bloodstream infection sepsis using single-cell RNA sequencing

**DOI:** 10.3389/fimmu.2024.1380211

**Published:** 2024-06-05

**Authors:** Shengwei Zhang, Nan Zhang, Jing Han, Zeyu Sun, Hua Jiang, Wenhua Huang, Decong Kong, Qian Li, Yuhao Ren, Shishun Zhao, Yongqiang Jiang, Peng Liu

**Affiliations:** ^1^State Key Laboratory of Pathogen and Biosecurity, Beijing Institute of Microbiology and Epidemiology, Academy of Military Medical Sciences, Beijing, China; ^2^Department of Clinical Laboratory, Dongfang Hospital, Beijing University of Chinese Medicine, Beijing, China; ^3^College of Mathematics, Jilin University, Changchun, China

**Keywords:** *Klebsiella pneumoniae*, sepsis, bloodstream, PBMCs, scRNA-seq, immune state

## Abstract

**Background:**

*Klebsiella pneumoniae* is a common Gram-negative bacterium. Blood infection caused by *K. pneumoniae* is one of the most common causes of human sepsis, which seriously threatens the life of patients. The immune status of peripheral blood mononuclear cells (PBMCs) based on single-cell RNA sequencing (scRNA-seq) in acute stage and recovery stage of sepsis caused by *K. pneumoniae* bloodstream infection has not been studied.

**Methods:**

A total of 13 subjects were included in this study, 3 healthy controls, 7 patients with *K. pneumoniae* bloodstream infection in the acute stage (4 patients died), and 3 patients in the recovery stage. Peripheral blood of all patients was collected and PBMCs were isolated for scRNA-seq analysis. We studied the changes of PBMCs components, signaling pathways, differential genes, and cytokines in acute and recovery stages.

**Results:**

During *K. pneumoniae* acute infection we observed a decrease in the proportion of T cells, most probably due to apoptosis and the function of T cell subtypes was disorder. The proportion of monocytes increased in acute stage. Although genes related to their phagocytosis function were upregulated, their antigen presentation capacity-associated genes were downregulated. The expression of IL-1β, IL-18, IFNGR1 and IFNGR2 genes was also increased in monocytes. The proportion of DCs was depleted during the acute stage and did not recover during sepsis recovery. DCs antigen presentation was weakened during the acute stage but recovered fast during the recovery stage. pDCs response to MCP-1 chemokine was weakened, they recovered it quickly during the recovery stage. B cells showed apoptosis both in the acute stage and recovery stage. Their response to complement was weakened, but their antigen presentation function was enhanced. The proportion of NK cells stable during all disease’s stages, and the expression of IFN-γ gene was upregulated.

**Conclusion:**

The proportion of PBMCs and their immune functions undergo variations throughout the course of the disease, spanning from the acute stage to recovery. These findings provide new insights into the mechanism of PBMCs immune function during *K. pneumoniae* bloodstream infection sepsis and recovery and sets the basis for further understanding and treatment.

## Introduction

Sepsis is an inflammatory clinical syndrome caused by systemic infection. It can result in severe immune system dysfunction, leading to drastic changes in cytokines, and causing multi-system organ failure, which seriously endangers the life and health of patients (1, 2). With a high mortality rate in hospitals worldwide, the complexity and severity of sepsis is a recognized problem globally (3). Despite extensive efforts by researchers to enhance the cure rate of sepsis patients, no targeted treatment for sepsis has been developed as of now (4). Therefore, a clear understanding of the immune factors associated with the progression of sepsis is of great significance for the development of new treatment options to improve the cure rate of sepsis.

Bloodstream infections are one of the most common causes of sepsis in humans (5). Among known infectious agents, Gram-negative bacteria are the major contributing factor, accounting for about 60% of blood culture-positive bacteria in sepsis patients (6). Therefore, it is necessary to study the secondary sepsis after bloodstream infection caused by Gram-negative bacteria. *K. pneumoniae* is the main Gram-negative bacteria causing bloodstream infections in sepsis, second only to *E. coli* (7). The immune status of peripheral blood mononuclear cells (PBMCs) in acute stage and recovery stages of sepsis caused by *K. pneumoniae* bloodstream infection has not been studied based on single-cell RNA sequencing (scRNA-seq). PBMCs is an important immune defense barrier in the body. The study on the changes of PBMCs components and functions has guiding significance for the treatment and recovery strategy of *K. pneumoniae* sepsis patients.

In recent years, scRNA-seq has developed rapidly and has been applied to a variety of disciplines. This method makes it possible to accurately characterize cell types and their associated gene expression profiles in mixtures (8). Previous bulk RNA sequencing has provided limited insights into the pathogenesis of sepsis (9).

In this study, we discussed the dynamic changes of PBMCs immune function in patients with sepsis caused by *K. pneumoniae* bloodstream infection in different disease courses from the perspectives of cell composition, gene function, signaling pathway, and cytokine changes, providing a certain basis for further understanding and treatment. Our work will contribute to a deeper understanding of the role of PBMCs during *K. pneumoniae* bloodstream infection sepsis.

## Materials and methods

### Ethics approval

This study was approved by the Ethics Committee of Dongfang Hospital of Beijing University of Chinese Medicine (JDF-IRB-2022000101), and each patient signed the relevant informed consent form.

### Patient inclusion and exclusion criteria

The patients in this study were all from the wards and Emergency department of Dongfang Hospital, Beijing University of Chinese Medicine. Patients with *K. pneumoniae* bloodstream infection sepsis in the acute stage (KPN_ACU) met the following criteria: 1. Blood culture positive for *K. pneumoniae*; 2. Third International Consensus Definitions of Sepsis and Septic Shock (Sepsis 3.0) (10); 3. Body temperature > 38°C; 4. Sequential Organ Failure Assessment (SOFA) score ≥ 2. Patients with *K. pneumoniae* bloodstream infection sepsis in the recovery stage (KPN_REC) met the following criteria: 1. Body temperature < 37°C for 24 consecutive hours; 2. SOFA score < 2. The healthy control (HC) group was comprised of patients from the Physical Examination Department of Dongfang Hospital of Beijing University of Chinese Medicine, that met the following criteria: 1. No infectious diseases; 2. Normal liver and kidney function; 3. Blood counts and inflammatory markers are normal.

### Sample collection

Three different types of vacuum blood collection tubes (Greiner VACUETTE) were used for whole blood collection: 2ml EDTA (ethylenediaminetetraacetic acid) anticoagulant tube, 3ml sodium citrate anticoagulant tube and 3ml serum tube. All venous blood was collected on an empty stomach. In the HC group, blood was retrieved from fasting patients in the morning. Whole blood collection in the KPN_ACU group was performed within 24 hours after diagnosis of *K. pneumoniae* bloodstream infection sepsis, while in the KPN_REC group blood was collected within the first 24 hours during the recovery stage. The EDTA anticoagulant whole blood was immediately subjected to complete blood count and PBMCs isolation. The sodium citrate anticoagulant whole blood was centrifuged for 1500g for 10 mins and plasma was separated for D-dimer detection. The whole blood collected from the serum tube was centrifuged for 2000g for 10 mins and serum was separated and stored in the refrigerator (Haier Biomedical) at -80°C for later use.

### Cell count, D dimer, inflammation cytokines detection

Whole blood in the 2ml EDTA anticoagulant vacuum blood collection tube was inverted five times, and a complete blood count was performed by automatic hematology analyzer XN-2000 (Sysmex, Japan). D-dimer was detected in the plasma isolated from 3ml sodium citrate anticoagulant whole blood using the automatic coagulation analyzer CS 5100 (Sysmex, Japan). Serum C-reactive protein (CRP) and procalcitonin (PCT) were measured using the automatic electrochemical luminescence immunoanalyzer Roche 801 (Roche, Switzerland).

### PBMCs isolation

PBMCs were isolated from fresh EDTA anticoagulant whole blood samples using density-gradient centrifugation, following a previously established protocol (11). Briefly, whole blood was transferred to 50 mL centrifuge tubes (Cornig, USA) and diluted approximately 1:1 with 1×PBS at room temperature. The mixture was then layered on top of Ficoll-Paque Plus (GE Healthcare, USA) and centrifuged at 500g for 30min. The PBMCs layer obtained was transferred to a fresh 50 mL centrifuge tube and washed three times with 10ml RPMI-1640 (Gibco, USA). Cell viability and count were confirmed using an Automated Cell Counter TC20 (BIO-RAD, USA). The criteria for sample inclusion were a minimum of 1×10^6^ per sample and a cell viability exceeding 90%. Only preparations with high cell viability were subjected to sequencing library preparation.

### Single-cell RNA-seq of PBMCs

Single PBMC cells were captured using a 10x Chromium Controller (10x Genomics, USA) and libraries were prepared following the guidelines provided in the Single Cell 3’ Next GEM V3.1 Reagent Kits User Guide (10x Genomics, USA). The captured PBMCs were loaded onto a Chromium Single Cell Chip (10x Genomics) as per the manufacturer instructions facilitating co-encapsulation with barcoded Gel Beads at a target capture rate of 8000 individual cells per sample. Once into the chip, captured cells were lysed and the released RNA was barcoded through reverse-transcription in individual single-cell gel beads within the emulsion (GEMS). Within each droplet, complementary DNA (cDNA) was generated and amplified through reverse-transcription on a T100 PCR Thermal Cycler (Bio-Rad, USA). Subsequently, cDNA concentration and quality were assessed using Qubit Fluorometer (Thermo Scientific, USA) and bioanalyzer 2100 (Agilent, USA), respectively. Following the manufacturer’s recommendations, scRNA-seq libraries were constructed and sequenced to a depth of 30,000-60,000 reads per cell on a NovaSeq 6000 platform (Illumina, USA).

### Single-cell RNA-seq data processing

The raw data was initially processed using Cell Ranger version 3.0.2, converting the BCL file into a fastq file. The feature barcode matrix was generated using the human genome reference version GRCh38. Then, we conducted data quality control with R software DoubletFinder (v.3.5.1) considering indicators such as Total Unique Molecular Identifier (UMI) counts, number of detected genes, expression rate of hemoglobin genes, expression rate of mitochondrial genes, and the minimum cells per gene. Cells meeting the following criteria were included in the analysis: 1. UMI counts less than 25,000; 2. detected genes between 500 and 5,000 (500 < nFeature_RNA < 5000) ; 3. less than 20% of mitochondrial genes; 4. less than 1% of hemoglobin genes; 5. minimum cells per gene>10. After completing the quality control process, low quality cells were removed, resulting in a data set consisting of higher-quality single-cell data (12, 13).

### Dimensionality reduction and clustering

‘IntegrateData’ function from the Seurat3 package in R (v.3.5.1) was used to eliminate batch effects between samples (14). The primary approach involved dimensionality reduction through Canonical Correlation Analysis (CCA), followed by subspace cell computation based on the Mutual Nearest Neighbors (MNN) algorithm, and finally, anchoring. Highly variable genes were identified using the ‘FindVariablesFeatures’ function also in the Seurat3 package. A principal component analysis (PCA) was performed using the ‘RunPCA’function to reduce the dimensions that would be used in subsequent analysis, and Elbow Point was used for selection (9). The Elbow Point diagram was generated using a heuristic evaluation method, and the 9th principal component was determined as the inflection point. Finally, the Seurat3 package’s ‘RunUMAP’ function was used to further reduce and visualize the PCA results. The number of cell clusters in each case was calculated at a resolution of 0.2, resulting in a total of 14 clusters.

### Cell type annotation

The Seurat3 package’s ‘FindAllMarkers’ function was employed to identify markers for each cell cluster (15). Cluster annotation was performed using typical markers associated with specific cell types. To validate cluster annotations, we utilized the R package SingleR and the human BlueprintEncodeData dataset. This involved comparing the transcriptome of each single cell with the reference dataset to determine cell identity. In a two-step process, we first used the R package SingleR for machine annotation. Subsequently, manual annotation was performed based on to the Cell Maker website. The final classification resulted in five main cell clusters: T cells, B cells, NK cells, monocytes, and dendritic cells (DCs). Each cell type was further divided into multiple subtypes.

### Differentially expressed genes identification

The ‘FindMarkers’ function in R package Seurat3 was employed to analyze DEGs between distinct cell groups, and the *p*-value were adjusted using the Bonferroni correction method. Significantly DEGs were identified based on an adjusted *p*-value < 0.05. The statistical method used for analyzing DEGs in different groups of immune cells was Wilcoxon sign rank test.

### GO enrichment analysis

DEGs derived from experimental data were mapped genes to Gene Ontology (GO) (16). The enrichment of each GO entry in the gene list was calculated by comparing the ratio of the observed number of genes to the expected number based on the random distribution of the background genome, using Fisher precision tests in SingleR. To control for multiple comparisons and reduce false positive results, Bonferroni correction was applied in SingleR.

### Pathway analysis and module scores

We conducted a comprehensive pathway analysis for all major immune cell types employing Ingenuity Pathway Analysis (IPA). DEGs (False Discovery Rate [FDR] < 0.01) for each cell type were enriched. Using multiple manually curated pathways and FDR values, we performed a pathway activity analyses to assess whether significantly enriched pathways (FDR < 0.01) were activated or inhibited. Pathway module scores were determined for each cell type by aggregating DEGs across all groups (17). We utilized gene formation modules within DEGs, classified by biological processes or pathways of interest in GO databases, and select module genes based on actual enriched GO processes. The pathway module scores were calculated, and the significance of the scores for different modules was tested through the ‘AddModuleScore’ function of the Seurat3 package (18).

### Cytokines, chemokines, complement and Superoxide Dismutase (SOD) detection

Stored serum samples were retrieved from the -80°C refrigerator and allowed to thaw at room temperature. Human inflammatory cytokines and chemokines were determined using a Cytokine/Chemokine/Growth Factor Convenience 45-Plex Human Panel 1 (Thermo Fisher, USA) with Luminex xMAP platform (Thermo Fisher, USA). Complement components C1q, C3 and B Factor were detected using the automated biochemical analyzer Cobas 701 (Roche, Switzerland). The assessment of SOD levels was performed using a colorimetric detection kit (Ruiyuan, China).

### Statistical analysis

Continuous variables that fit the normal distribution are represented by mean and standard deviation (SD). Continuous variables that do not fit the normal distribution are represented by median and interquartile spacing. Categorical variables are represented by frequency and proportion. Continuous variables between the two groups were compared using either the Student’s t test or the non-parametric Mann-Whitney test. 2-sided *p*-value < 0.05 were considered statistically significant using Graphpad prism software, version 9.0.

## Results

### Basic clinical data of patients with *K. pneumoniae* bloodstream infection sepsis

**Figure 1:**
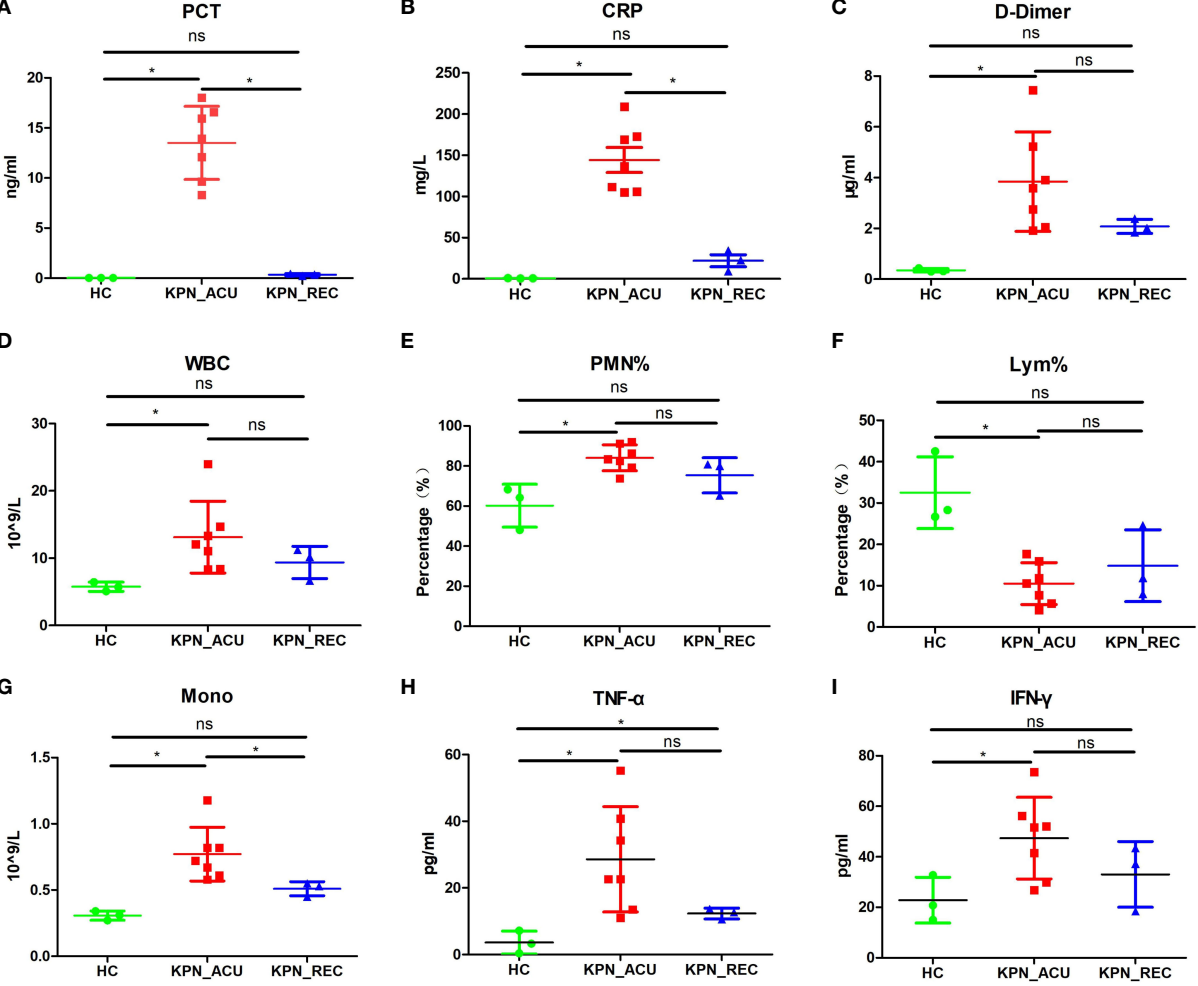
The changes of the indicators in the peripheral blood of the subjects between the three groups of patients. **(A)** Inflammatory marker of procalcitonin (PCT) in serum of three groups. **(B)** Inflammatory marker of C-reactive protein (CRP) in serum of three groups. **(C)** Coagulation marker of D-dimer in peripheral blood plasma in three groups. **(D)** White blood cell (WBC) count in three groups. **(E)** Polymorphonuclear leukocyte percentage (PMN%) in three groups. **(F)** Lymphocyte percentage (Lym%) in three groups. **(G)** Monocyte count (Mono) in three groups. **(H)** Serum levels of TNF-α in three groups. **(I)** Serum levels of IFN-γ in three groups. Each point represents a sample. Significance was determined using Mann-Whitney test, where *p-value < 0.05 indicates statistical significance.

To investigate the dynamic immune response of PBMCs in patients with sepsis caused by *K. pneumoniae* bloodstream infection, we assembled three distinct groups for the study. The *K. pneumoniae* acute stage group (KPN_ACU) comprised seven patients, aged 69-91 years, with positive blood cultures of *K. pneumoniae*. Among them, four women, and unfortunately, four patients in this group did not survive. For the *K. pneumoniae* recovery group (KPN_REC), which involved a follow-up of the acute stage group, 3 patients, aged 69-80 years (2 women), successfully recovered(8 to 15 days after determining KPN_ACU). Additionally, we included a healthy control group (HC) consisting of 3 individuals, aged 67-88 years (2 women), selected from the physical examination department. Peripheral blood and serum samples were collected from patients in the KPN_ACU and HC groups, with one sample per patient. For patients in the KPN_REC group, two sets of samples were collected: one during the acute infection stage and another during the recovery stage. Basic clinical data of the patients are provided in **Supplementary Table 1**. The collected samples underwent follow-up scRNA-seq, analysis of inflammatory indicators, and assessment of immune indicators to comprehensively study the immune response dynamics. First, we evaluated eight sepsis-associated indicators in the serum of patients and compared the results across different groups. The findings revealed significant differences among the groups. In comparison to the HC group, the KPN_ACU group, exhibit significantly elevated levels of PCT (*p* < 0.05) (**Figure 1A**), C-reactive protein ([CRP], *p* < 0.05) (**Figure 1B**), D-dimer (*p* < 0.05) (**Figure 1C**), white blood cell count ([WBC], *p* < 0.05) (**Figure 1D**), polymorphonuclear leukocyte percentage ([PMN%], *p* < 0.05) (**Figure 1E**), monocyte count ([Mono], p < 0.05) (**Figure 1G**), tumor necrosis factor alpha ([TNF-α], *p* < 0.05) (**Figure 1H**), and interferon-gama ([IFN-γ], *p* < 0.05) (**Figure 1I**), along with a significant decrease in lymphocyte proportion ([Lym%], *p* < 0.05) (**Figure 1F**). When comparing KPN_REC with KPN_ACU, a notable reduction was observed in the levels of CRP (*p* < 0.05) and PCT (*p* < 0.05) and Mono (p < 0.05), indicating a positive response to treatment. Other indicators such as D-dimer (*p* = 0.12), WBC (*p* = 0.27), PMN% (*p* = 0.18), TNF-α (*p* = 0.12) and IFN-γ (*p* = 0.21) showed a decreasing trend, while Lym% exhibited an increasing trend, though statistical significance varied. Importantly, when comparing KPN_REC with HC, there were no statistically significant differences in inflammation markers (CRP, PCT, IFN-γ), except for TNF-α, coagulation marker (D-dimer), and blood routine markers (WBC, PMN%, Lym%, Mono) in KPN_REC (*p* > 0.05) (**Figure 1**).

The above results indicate that the patients with *K. pneumoniae* bloodstream infection sepsis are predominantly elderly, facing high mortality rates, and experiencing significant alterations in immune cell proportions and inflammatory responses. The elevated D-dimer indicates abnormal blood clotting function, potentially leading to thrombosis. During the recovery stage, there is a significant decrease in inflammatory factors, thrombus levels, SOFA score, and body temperature, accompanied by a tendency for the immune cell proportion to return to normal. However, it is noteworthy that the recovery of inflammatory factors is notably faster than the restoration of the immune cell proportion. This suggests that there may still be some imbalance or disorder in the composition of immune cells among patients during the recovery stage.

### Significant changes in the composition of PBMCs in patients with sepsis caused by *K. pneumoniae* bloodstream infection

**Figure 2:**
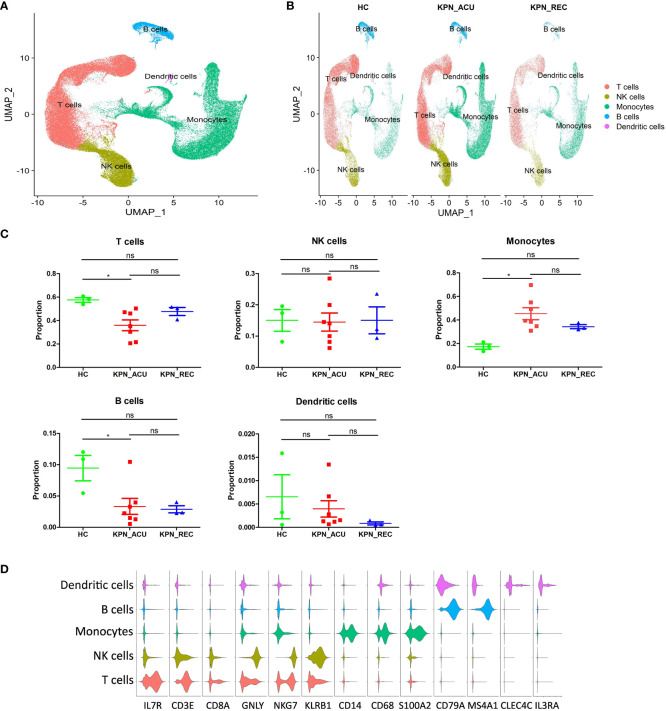
Cell type identification and proportion change were based on scRNA-seq of PBMCs. **(A)** UMAP presentation of T cells (red), NK cells (yellow), Monocytes (green), B cells (blue) and dendritic cells (pink) in PBMCs of all samples. **(B)** UMAP presentation of T cells (red), NK cells (yellow), Monocytes (green), B cells (blue) and dendritic cells (pink) after integration among the three groups. **(C)** Proportions of the T cells, NK cells, Monocytes, B cells and dendritic cells in the HC, KPN_ACU and KPN_REC groups. Significance was determined using Mann-Whitney test. *p < 0.05. **(D)** Violin plots of17 canonical annotation marker genes (columns) for different cell types (rows).

Next, the PBMCs isolated from the blood of patients in the three study groups (HC, n = 3; KPN_ACU, n = 7; KPN_REC, n = 3) were subjected to scRNA-seq. We collected 122,374 high-quality cells, with an average of 9,413 cells per sample. After strict quality control, all samples’ cells were integrated following batch effect correction. They were then analyzed using unbiased clustering and uniform manifold approximation and projection (UMAP). All clusters were composed of cells from each sample, indicating a successful batch effect correction. We identified 14 clusters with distinct transcriptomic signatures and annotated cells based on canonical annotation marker genes of immune cells. These were defined as five typical immune cell types: T cells (CD3E^+^CD8A^+^IL7R^+^), NK cells (GNLY^+^NKG7^+^KLRB1^+^), monocytes (CD14^+^CD68^+^S100A2^+^), B cells (CD79A^+^MS4A1^+^), and DCs (CLEC4C^+^IL3RA^+^). The violin plots show good differentiation of different cells (**Figures 2A, B, D**). We observed changes in the proportion of various cell types among the three study groups, revealing dynamic changes during disease progression. Compared with HC group, the proportion of T cells significantly decreased in KPN_ACU group and slightly decreased in KPN_REC group. However, the proportion of T cells in KPN_REC group was slightly higher than that in KPN_ACU group. The proportion of NK cells was similar among the three groups. Monocytes in KPN_ACU group increased significantly and showed a trend of recovery in KPN_REC group. The proportion of B cells in KPN_ACU group significantly decreased, and remained not at a lower level in KPN_REC group. The proportion of DCs was also similar among the three groups (**Figure 2C**).

The results above evidence that *K. pneumoniae* bloodstream infection caused significant interference in the proportions of PBMCs. Different types of immune cell may be dysfunctional in both acute infection and recovery stages.

### T cells were characterized by apoptosis and exhaustion, and CD4+ T cells and CD8+ T cells exhibit different functions in acute infection stage

**Figure 3:**
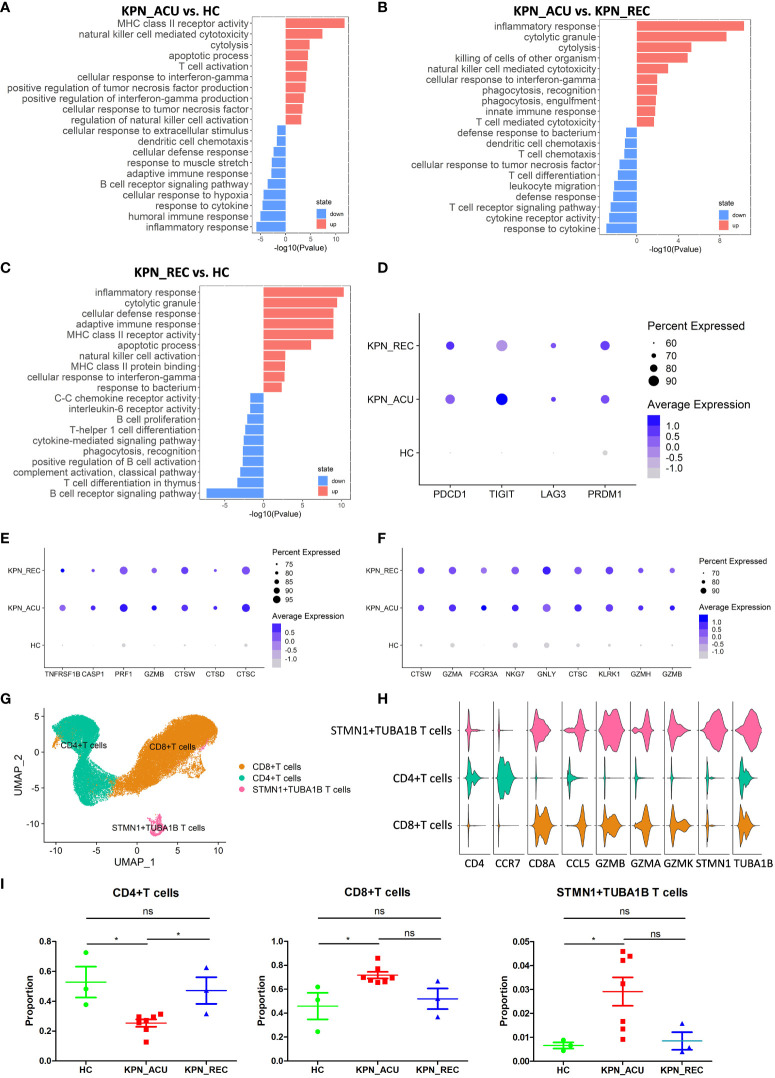
T cells were characterized by apoptosis and exhaustion and the composition and function of T cells subtypes appeared altered during sepsis. **(A)** Global transcriptome differences between KPN_ACU and HC groups were evaluated in T cells by GO enrichment analysis of upregulated (red) and downregulated (blue) biological processes (y-axe). **(B)** GO enrichment analysis between KPN_ACU and KPN_REC groups. **(C)** GO enrichment analysis between KPN_REC and HC groups. **(D)** Bubble plot shows the expression levels of exhaustion-related genes of T cells in three groups. **(E)** Bubble plot shows the expression levels of apoptosis-related genes of T cells in three groups. **(F)** Bubble plot shows the expression levels of cytotoxicity-related genes of T cells in three groups. **(G)** UMAP presentation of three T cells subtypes. **(H)** Violin plots of canonical annotation marker genes (columns) for different subtypes (rows). **(I)** Proportions of the three subtypes. ns, no statistical significance; *P < 0.05.

T cells, important immune cells in the body against bacterial infection, exhibited a decreased proportion during the acute stage of *K. pneumoniae* bloodstream infection sepsis and showed a recovery trend in recovery stage (**Figure 2C**). Therefore, the changes in T cell composition and immune function during the whole disease stage may reflect the severity of the disease and the progress of recovery. To further understand the biological functional changes involved in T cells response to *K. pneumoniae* sepsis, we performed a Gene Ontology (GO) enrichment analysis revealing differences in the T cells transcriptome between the three groups of study. Compared to the HC group, T cells from the KPN_ACU group exhibited upregulation in the antigen presentation and cytotoxicity pathways, indicating the activation of the bacterial defensive function of T cells (**Figure 3A**). On the other hand, the biological processes regarding response to cytokine, humoral immune response and inflammatory response are downregulated, suggesting that T cells are in immunosuppressant state at the same time (**Figure 3A**). Compared with the KPN_REC group, genes related to cytolytic granule production and T cell mediated cytotoxicity were upregulated in the T cells from KPN_ACU patients, while functions such as leukocyte migration and dendritic cell chemotaxis are downregulated (**Figure 3B**). This indicates that the cytotoxicity of T cells was enhanced during the acute infection stage, but their auxiliary immune function was inhibited to a certain extent. Nevertheless, with the recovery of patients, the auxiliary immune function also gradually recovered. Compared to the HC group, the cytolytic granule production, apoptotic process, MHC class II receptor activity, inflammatory response, and adaptive immune response of T cells from the KPN_REC group were upregulated, while the cytokine-mediated signaling pathway and C-C chemokine receptor activity were downregulated (**Figure 3C**), indicating that the patients were gradually recovering, but not fully recovered.

We also studied the expression of immune function genes in T cells and found that T cell exhaustion-related markers, including PDCD1, TIGIT, LAG3, and PRDM1, were significantly upregulated in the KPN_ ACU group compare to T cells from HC group. However, these markers were not downregulated in the T cells from the KPN_ REC group (**Figure 3D**), indicating that the T cell exhaustion that occurred during acute infection did not fully recover during the recovery stage. The apoptosis-related markers TNFRSF1B, CASP1, PRF1, GZMB, CTSW, CTSD and CTSC were significantly upregulated in the T cells from both KPN_ ACU and KPN_REC groups compared to HC group, indicating that T cell apoptosis, which occurred during acute infection, did not fully recover during the recovery stage (**Figure 3E**). These findings support our previous observation of a lower proportion of T cells in the KPN_REC group compared to the HC group. The immunosuppression of sepsis mainly manifests as lymphocyte apoptosis and exhaustion, especially T cells (19), as confirmed by the increased expression of apoptosis related genes. Similarly, T cell cytotoxicity markers CTSW, GZMA, GZMB, FCGR3A, NKG7, GNLY, CTSC, KLRK1, and GZMH were found significantly upregulated in both KPN_ ACU and KPN_REC groups (**Figure 3F**), indicating a significant increase in T cell killing ability during acute infection stage that persists into the recovery stage.

We performed a subpopulation cluster analysis of T cells to study their dynamics at a finer resolution. Furthermore, we clustered the T cells into three subpopulations: CD4+ T cells (CD4^+^CCR7^+^), CD8+ T cells (CD8A^+^GZMA^+^), and STMN1+TUBA1B T cells (STMN1^+^TUBA1B^+^) (**Figures 3G, H**), to study their dynamics at a finer resolution. We observed a significant decrease in the proportion of CD4+ T and a simultaneous increase in the proportion of CD8+ T cells in the KPN_ACU group compared to the HC group (**Figure 3I**). This suggests that the overall decrease in T cell proportion was primarily attributable to the decrease in CD4+ T cells. CD4+ T cells are helper T cells, whose main function is to secrete cytokines, chemokines, and recruit target cells (20). The decrease of CD4+ T cells proportion in KPN_ACU group indicates that the ability of the body to rapidly initiate immune response is reduced, and the immunosuppressive state of CD4+ T cell subtypes is the main cause of T cell immunosuppression. CD8+ T cell is a cytotoxic T cell, whose main function is to clear cells infected by pathogens (19). The proportion of CD8+ T cells in KPN_ACU group increased (**Figure 3I**), indicating that there were more infected cells. The decrease of CD4+/CD8+ T cells ratio indicates that the patient’s immune function is decreased. During the recovery stage, the killing ability was still high, and the killing ability was mainly derived from CD8+ T cells, which was consistent with the increase in the proportion of CD8+ T cells (**Figures 3F, I**). Genes related to MHC-II antigen presentation were significantly upregulated in T cells from KPN_ACU group. Although these genes were downregulated in T cells from individuals in the KPN_REC group, their expression levels remained higher than those in T cells recovered from the HC group. MHC-II antigen presentation genes were upregulated mainly in CD8+ T cells (**Supplementary Figures S1A–C**). These results indicate that CD8+ T cells play a more important role in antigen recognition of *K. pneumoniae* infection defense. STMN1+TUBA1B T lymphocytes also highly expressed CD8A and GZMB markers, indicating that STMN1+TUBA1B T cells were cytotoxic. STMN1+TUBA1B T cells accounted for a low proportion of T cells, that was found significantly higher in the KPN_ACU group, decreasing to the level of the HC group in the KPN_REC group (**Figure 3I**). Detection of STMN1+TUBA1B T cells may be able to predict recovery from sepsis. STMN1+TUBA1B T cells have downregulated biological processes of antigen presentation and B cells activation during acute stage compared to HC group (**Figure S1D**; **Supplementary Table 2**), indicating that STMN1+TUBA1B T cells have different functions to CD8+ T cells and CD4+ T cells, but their specific functions need further study.

In summary, T cells were characterized by apoptosis and exhaustion, and the helper function of CD4+ T cells was decreased, and the cytotoxicity and antigen presentation function of CD8+ T cells were enhanced.

### Monocytes phagocytosis was upregulated, but antigen presentation was downregulated in acute infection patients

**Figure 4:**
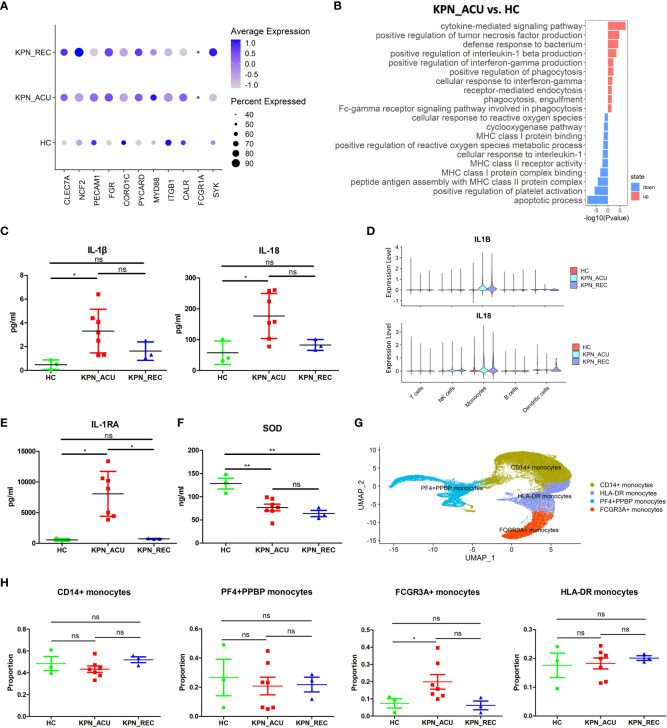
Monocytes phagocytosis and multiple cytokine genes were up-regulated in KPN_ACU group. **(A)** Bubble plot shows the expression levels of phagocytosis genes of Monocytes in three groups. **(B)** GO enrichment analysis of upregulated and downregulated biological processes in monocytes between KPN_ACU and HC groups. **(C)** Inflammatory marker of IL-1β (left) and IL-18 (right) in serum of three groups. **(D)** Violin plots of IL1B (up) and IL18 (down) genes in five cells. **(E)** Inflammatory suppressor marker of IL-1RA in serum of three groups. **(F)** Serum SOD content in three groups. **(G)** UMAP presentation of four Monocytes subtypes. **(H)** Proportions of the four subtypes in three groups. ns, no statistical significance; *P < 0.05, **P<0.01.

Monocytes are the largest immune cells in peripheral blood, which have important immune functions such as phagocytosis and antigen presentation (17). Compared with their counterparts in the HC group, the proportion of monocytes in KPN_ACU group was significantly higher. Although the proportion of monocytes in KPN_REC group was lower, it also remained slightly higher than that in HC group (**Figure 2C**). Additionally, the proportion of monocytes lowered with the decrease of inflammatory indicators (**Figures 1**, **2C**), suggesting that monocytes are an important part of the body’s defense system against *K. pneumoniae*. DGEs analysis showed that compared with HC group, the expression of genes related to phagocytosis, such as CLEC7A, NCF2, CD14, PECAM1, FGR, CORO1C, PYCARD, MYD88, ITGB1, CALR, FCGR1A and SYK, was increased in the monocytes from the KPN_ACU group (**Figure 4A**), indicating that the monocytes phagocytosis function of the patient is activated after infection with *K. pneumoniae*. These results were further supported by the GO enrichment analysis that showed an upregulation of the phagocytosis pathway in monocytes from the KPN_ACU group (**Figure 4B**). On the other hand, the expression of the phagocytosis genes was decreased in KPN_REC group compared to KPN_ACU group but still higher than that in the HC group (**Figure 4A**). Furthermore, the phagocytosis pathway (positive regulation of phagocytosis) was also observed to be downregulated in the KPN_REC group compared to the KPN_ACU group, but not as much as in the HC group (**Figure 4A**; **Supplementary Figures S2A, B**).

The levels of interleukin-1β (IL-1β) interleukin-18 (IL-18) and interleukin-1 receptor antagonist (IL-1RA) in serum were significantly increased in KPN_ACU group compared to the HC group and, in the case of IL-1RA, also compared to the KPN_REC group (**Figures 4C, D, E**). The PBMCs scRNA-seq data showed that IL-1β, IL-18 and IL-1RA genes (IL1B, IL18, IL1RN) were mainly expressed in monocytes (**Supplementary Figures S2C, D, E**), indicating that among the PBMCs, IL-1β, IL-18 and IL-1RA in infected patients were mainly produced by monocytes in PBMCs. Similarly, compared to the HC group, IL-1β, IL-18 and IL-1RA genes in KPN_ACU and KPN_REC were significantly upregulated (**Figure 4D**; **Supplementary Figures S2D, E**), indicating that patients in acute stage had obvious inflammatory response, while patients in recovery stage had not fully recovered. IL-1RA is an immunosuppressive cytokine that inhibits IL-1β activity by binding to IL-1R1 (21), indicating that the anti-inflammatory genes of monocytes are also activated, and there is an interaction of pro-inflammatory and anti-inflammatory functions in the patient. In KPN_ACU group, IL-1β content in serum was significantly increased (**Figure 4C**), which could promote the antigen presentation ability of APC such as monocytes (22).The expression of monocytes antigen presentation genes was downregulated in the monocytes of the KPN_ACU group (**Supplementary Figure S2F**), and the GO enrichment analysis showed that multiple biological processes related to antigen presentation (peptide antigen assembly with MHC class II protein complex, MHC class I protein binding) were also downregulated (**Figure 4B**). This suggests that the ability of IL-1β to promote monocytes antigen presentation maybe inhibited by IL-1RA.The high expression level of TNF-α receptor TNFRSF1B in monocytes (**Supplementary Figure S2H**) suggests that TNF-α could induce the production of IL-1β in this cell type. In addition, TNF-α activates phospholipase A2 and releases superoxide, causing DNA breakage (23). Our study also found a downregulation of biological process that would inhibit the production of reactive oxygen species in patients from the KPN_ACU group compared to HC group (**Figure 4B**). Additionally, the serum levels of superoxide dismutase (SOD) decreased in individuals from both KPN_ACU and KPN_REC groups (**Figure 4F**), suggesting that the ability to remove oxygen free radicals is reduced in patients with sepsis. At present, it is believed that diffuse intravascular coagulation and toxic shock caused by Gram-negative bacteria are caused by excessive TNF-α production stimulated by bacterial endotoxin (24). Therefore, the increase of D-dimer content maybe caused by excessive TNF-α in KPN_ACU (**Figures 1C, H**).

To further investigate the immune function of monocytes during *K. pneumoniae* bloodstream infection, we split the monocytes population into four subtypes that represent different functions: CD14+ monocytes (CD14^+^), HLA-DR+ monocytes (HLA-DRA^+^HLA-DQB1^+^), PF4+PPBP+ monocytes (PF4^+^PPBP^+^), FCGR3A+ monocytes (FCGR3A^+^CDKN1C^+^) (**Figure 4G**). They were no significant differences in the proportion of these cells between the different study groups, except for a significant increase in the numbers of FCGR3A+ monocytes in the KPN_ACU group compared to HC group (**Figure 4H**). Compared with HC group, CD14+ monocytes from the KPN_ACU group were observed to highly express the inflammatory and cell activation markers S100A9 and S100A8, indicating that CD14+ monocytes maybe play a major inflammatory function during acute infection stage (**Supplementary Figure S2G**). Similarly, CD14+ monocytes from KPN_ACU group were found to have significantly upregulated pathways related to bacterial defense and cytokines (**Supplementary Table 3**). HLA-DR+ monocytes from the KPN_ACU group highly expressed antigen presentation-related markers MHC-II and CD74 (**Supplementary Figure S2G**), and the biological processes involved in multiple antigen processing and presentation monocytes were also significantly upregulated, evidencing their role as antigen presenting cells (**Supplementary Table 3**). As expected, the PF4+PPBP+ monocytes from individuals in the KPN_ACU group expressed the platelet-related markers PF4, PPBP (**Supplementary Figure S2G**), and the clotting pathway (hsa04610: Complement and coagulation cascades) and platelet activation were upregulated (**Supplementary Table 3**), evidencing that these monocytes are involved in coagulation or platelet immunity after *K. pneumoniae* infection. FCGR3A+ monocytes from the KPN_ACU group highly upregulated multiple lysosome biological processes, indicating that these cells mainly played a killing function (**Supplementary Table 3**).

In summary, monocytes are activated after *K. pneumoniae* infection, with increased cell proportion, enhanced phagocytosis function, and are able to interact with a variety of cytokines, and different subtypes play different functions. However, antigen presentation was downregulated. Therefore, monocytes play an important role in the defense of *K. pneumoniae*.

### The antigen presentation capabilities of DCs are downregulated in the acute stage of sepsis but rapidly recover during the recovery stage

**Figure 5:**
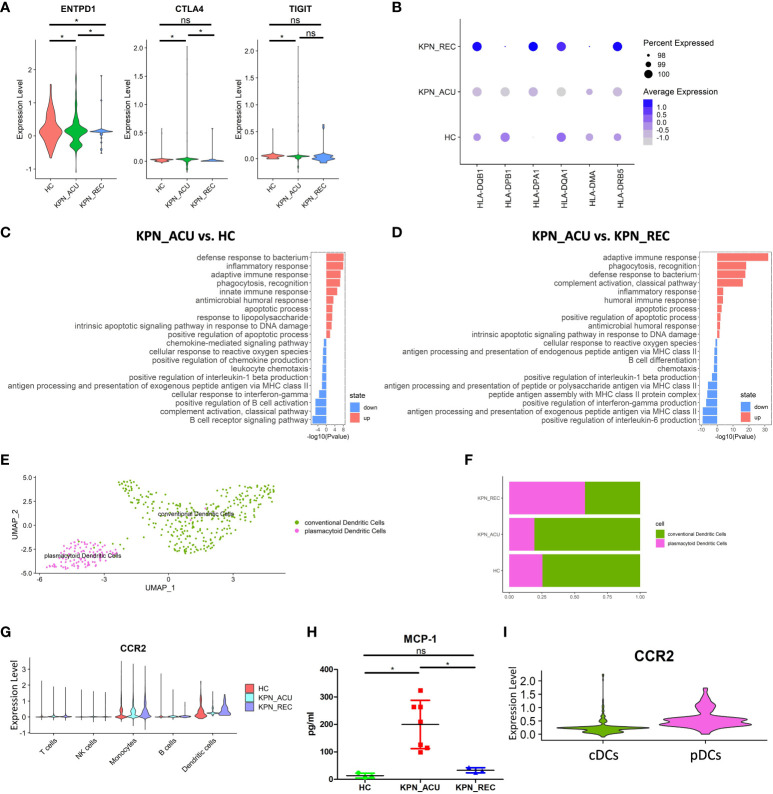
Changes in transcriptional function of DCs and its subtypes. **(A)** Violin plots of exhaustion genes of DCs in three groups. **(B)** Bubble plot shows the expression levels of antigen presentation genes of DCs in three groups. **(C)** GO enrichment analysis of DCs between KPN_ACU and HC groups. **(D)** GO enrichment analysis of DCs between KPN_ACU and KPN_REC groups. **(E)** UMAP presentation of two DCs subtypes. **(F)** Histogram showed the proportion of cDCs and pDCs in three groups. **(G)** Violin plots of CCR2 genes of five cells in three groups. **(H)** The level of MCP-1 in serum of three groups. **(I)** Violin plots of CCR2 genes of cDCs and pDCs. ns, no statistical significance; *P < 0.05.

DCs is the main antigen presentation cell type *in vivo*, and changes in their function affect the efficiency of pathogen recognition by immune cells (25). Our study did not find any significant difference in the proportion of DCs between the different study groups (**Figure 2C**). We suspected DCs may have changed primarily in function rather than proportions. So, we detected the expression of DCs exhaustion markers (ENTPD1, CTLA4, TIGIT). These genes were significantly upregulated in the KPN_ACU group (**Figure 5A**), which could explain a slight downward trend in cell proportion. In the KPN_REC group, the expression levels of exhaustion markers were lower than those found in the KPN_ACU group but still higher than the levels in HC group (**Figure 5A**). This indicates that DCs exhaustion may not fully recover during the recovery phase. Furthermore, DEGs analyses showed a downregulation of DCs genes (HLA-DQB1, HLA-DPB1, HLA-DPA1, HLA-DQA1, HLA-DMA, HLA-DRB5) related to antigen presentation in KPN_ACU group. This suggests that the antigen presentation capabilities of DCs from these individuals is probably decreased (**Figure 5B**), impacting the recognition of *K. pneumoniae*. The average expression of antigen presentation-related genes in the KPN_REC group was higher than that in HC and KPN_ACU groups (**Figure 5B**), indicating that the antigen presentation capacity of DCs recovered rapidly during the recovery stage, possibly overcompensating the low DCs proportion during infection. This was also supported by the GO enrichment analysis (**Figures 5C, D**). So overall, DCs antigen presentation is reduced and there is some immunosuppression in patients during the acute infection stage, but rapid recovery during the recovery stage.

We divided the DCs population into 2 subtypes, plasmacytoid DCs ([pDCs], CST3^+^MSRB1^+^) and conventional DCs ([cDCs], TNFRSF17^+^ IGHA2^+^) (**Figure 5E**; **Supplementary Figure S3A**). Compared with HC group, the proportions of pDCs and cDCs in KPN_ACU group were similar, but the proportions of pDCs in KPN_REC showed an increasing trend, while the proportions of cDCs showed a decreasing trend (**Figure 5F**; **Supplementary Figure S3B**). The average expression of antigen presentation-related genes is downregulated in both pDCs and cDCs from KPN_ACU group. The recovery rate of antigen presentation-related genes in cDCs was faster than that in pDCs (**Supplementary Figures S3C, D**).

Cell surface chemokine receptor 2 (CCR2) is highly expressed in DCs (26). CCR2 expression was downregulated in DCs during acute stage but upregulated during recovery stage (**Figure 5G**). The serum level of monocyte chemoattractant protein-1 ([MCP-1], a chemokine recognized by CCR2) was increased in KPN_ACU group but decreased to normal levels in KPN_REC group (**Figure 5H**). These results suggest that overproduction of MCP-1 during acute stage maybe compensate for the lack of CCR2. Further DEGs analysis specifically looking at each of the DCs subtypes revealed that the CCR2 gene is mainly expressed on pDCs (**Figure 5I**), and gets downregulated during acute stage (**Figure 5G**; **Supplementary Figure S3E**).

In conclusion, DCs has weakened antigen presentation in patients with *K. pneumoniae* bloodstream infection sepsis. pDCs response to MCP-1 chemokine is weakened in the acute stage. In the recovery stage, the recovery rate of antigen presentation is faster than the proportion of DCs (**Figures 2C**, **5B**).

### B cells activation is dysregulated during acute and recovery stage

**Figure 6:**
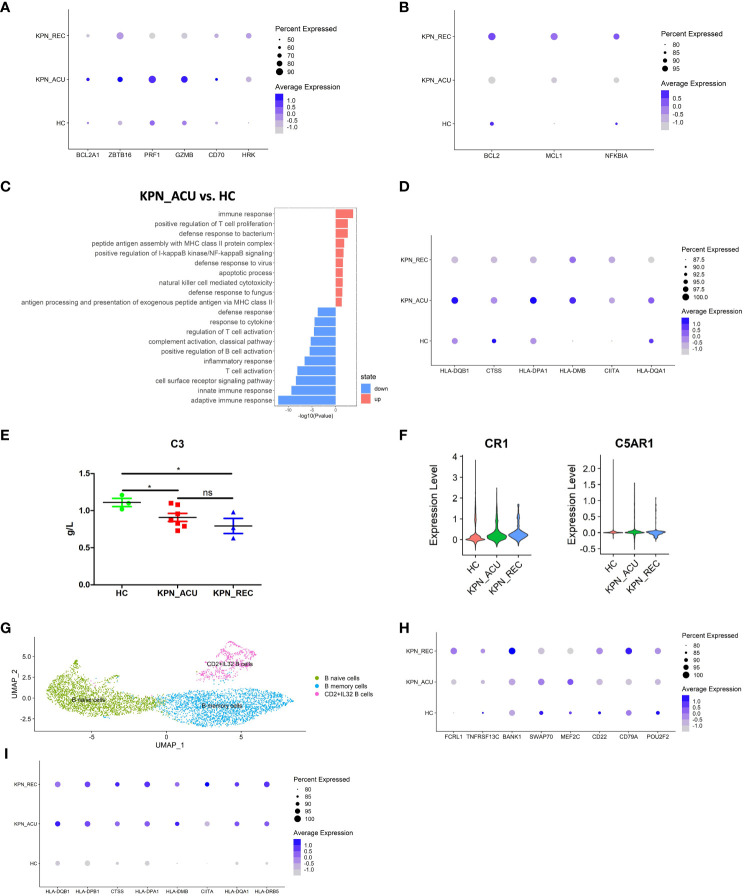
Changes in transcriptional function of B cells and its subtypes and the interaction with the complement. **(A)** Bubble plot shows the expression levels of apoptosis genes of B cells in three groups. **(B)** Bubble plot shows the expression levels of apoptosis inhibition genes of B cells in three groups. **(C)** GO enrichment analysis of B cells between KPN_ACU and HC groups. **(D)** Bubble plot shows the expression levels of antigen presentation genes of B cells in three groups. **(E)** The level of C3 in serum of three groups. **(F)** Violin plots of CR1 genes (left) and C5AR1 genes (right) of B cells in three groups. **(G)** UMAP presentation of three B cells subtypes. **(H)** Bubble plot shows the expression levels of activated genes of CD2+IL32 B cells in three groups. **(I)** Bubble plot shows the expression levels of antigen presentation genes of CD2+IL32 B cells in three groups.

B cells in PBMCs can be transformed into plasma cells secreting antibodies by pathogen stimulation, and participate in humoral immune response. B cells can also process and present antigens, and secrete cytokines such as interleukin, TNF, IFN, etc (27). B cell apoptosis was suspected based on the significantly low proportion of these cells during acute and recovery stages (**Figure 2C**). Correspondingly, the expression of apoptosis-related genes (BCL2A1, ZBTB16, PRF1, GZMB, CD70 and HRK) was increased during acute stage, while the expression levels of apoptosis inhibitors-related genes (BCL2, MCL1, NFKBIA) was observed to be decreased (**Figures 6A, B**). Furthermore, KEGG analysis showed that the apoptosis related pathway (hsa04210: apoptosis) was activated in the B cells during acute stage (**Supplementary Table 4**), and the GO enrichment analysis showed that the biological processes related to apoptosis were also upregulated in these cells (**Figure 6C**). This supports our suspicions of B cell apoptosis during *K. pneumoniae*-promoted sepsis, which may persist during the recovery stage. Furthermore, genes related to the production of interferon in B cells (MIX1, IFI6, IFITM3) were found to be downregulated in the KPN_ACU and KPN_REC groups compared the HC group (**Supplementary Figure S4A**). Compared with KPN_ACU group, the average expression of apoptosis genes was decreased and apoptosis inhibitor gene was increased (**Figures 6A, B**) and the apoptosis related pathway (hsa04210: apoptosis) was downregulated in KPN_REC group (**Supplementary Table 4**), but the cell proportion was similar to that of KPN_ACU (**Supplementary Figure 2C**), indicating that the recovery rate of apoptosis gene was faster than that of cell proportion. Antigen presentation is also an important immune function of B cells (28). On the contrary, the average expression of genes related to antigen presentation (HLA-DQB1, HLA-DQA1, HLA-DPA1, HLA-DMB, CIITA and CTSS) was increased in the B cells from KPN_ACU group (**Figure 6D**), indicating that the antigen presentation function of B cells was enhanced during acute stage. It is an important antigen presentation cell in the defense of *K. pneumoniae*.

CR1 is widely distributed on the surface of B cells, and its ligand is C3b, which is a regulator of B cell activation (29). On the other hand, the expression of complement receptor 1 (CR1) and complement receptor 5a (C5AR1) was found to be downregulated in the B cells during sepsis (**Figure 6F**), while the levels of complement 3 ([C3], **Figure 6E**), B factor and complement 1 q ([C1q], **Supplementary Figure S4B**) appeared decrease in the in serum of patients suffering acute stage too. Furthermore, this gene expression pattern and complement protein levels seems to remain low during recovery stage This suggests that complement activation is impaired in B cells during acute stage and continue to be damaged during recovery stage (**Supplementary Figure S4B**).

We divided B cells into three biologically relevant subtypes: B naive cells (TCL1A^+^IL4R^+^), B memory cells (AIM2^+^CD27^+^TNFRSF13B^+^), and CD2+IL32 B cells (CD2^+^IL32^+^) (**Figure 6G**; **Supplementary Figure S4C**). The proportion of the three cell subtypes did not change significantly between the three study groups. However, the CD2+IL32 B cells numbers showed a decreasing trend during acute stage and recovery stage (**Supplementary Figure S4E**). CD2+IL32 B cells is maybe a new type of B cell, which has not been reported yet. Even in depleting numbers, these cells displayed an upregulated expression of genes related to cell activation (FCRL1, TNFRSF13C, BANK1, SWAP70, MEF2C, CD22, CD79A, POU2F2) during acute stage (**Figure 6H**). The expression of some of those genes (FCRL1, TNFRSF13C, CD22, CD79A, POU2F2) continue to be upregulated during sepsis recovery stage too (**Figure 6H**). Similarly, the expression of antigen presentation-related genes (HLA-DQB1, HLA-DPB1, CTSS, HLA-DPA1, HLA-DMB, CIITA, HLA-DQA1, HLA-DRB5) was found to be upregulated in both KPN_ACU and KPN_REC groups (**Figure 6I**), with a higher antigen presentation module score in the recovery group (**Supplementary Figure S4D**). GO enrichment analysis showed that multiple biological processes of antigen presentation, such as antigen processing and presentation of exogenous peptide antigen via MHC class II, were upregulated in KPN_ACU group compared with HC group, and continue to be upregulated in the KPN_REC group (**Supplementary Figures S4F, G**), indicating that the antigen presentation function of CD2+IL32 B cells was activated, which maybe play an important role in the clearance of *K. pneumoniae*. These results demonstrate that CD2+IL32 B cells are important effector B cells. Unfortunately, we did not note plasma cells to analyze the antibody defense effect of *K. pneumoniae*.

In general, B cells were continuously decreased in KPN_ACU and KPN_REC groups, and interferon gene expression level and complement response were decreased, so defense function was decreased. However, a new B cell subtype has emerged, which has enhanced activation and antigen presentation function, and may play an important role in the recovery of *K. pneumoniae*.

### NK cells cytotoxicy- related genes are downregulated during acute sepsis while the IFN-γ gene expression is enhanced

**Figure 7:**
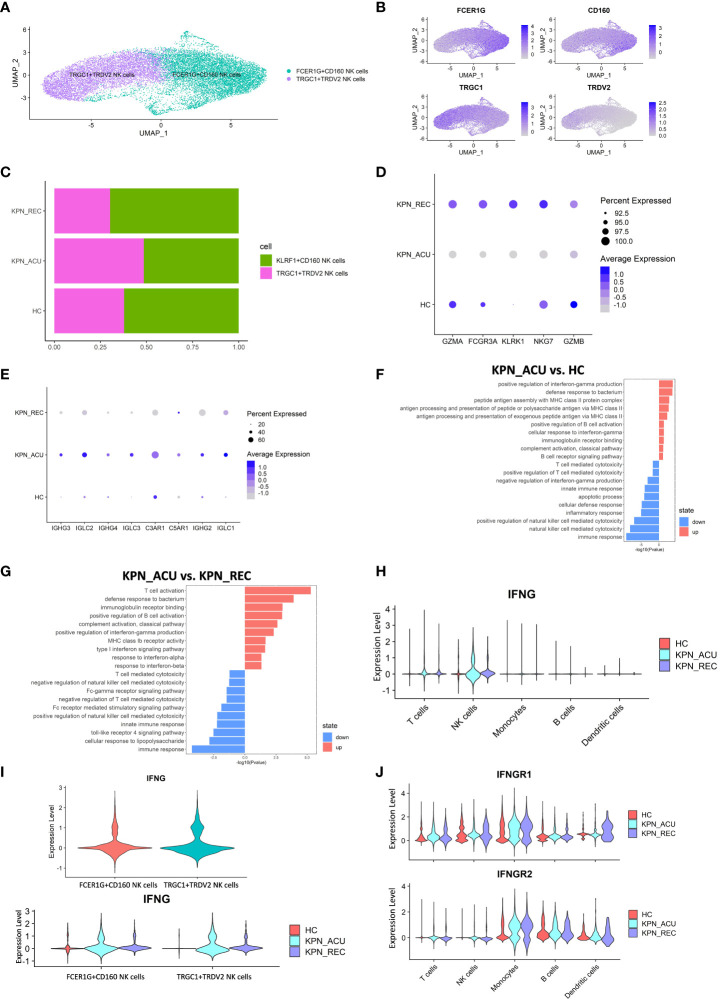
Cytotoxic function is reduced in NK cells but IFNG gene is upregulated. **(A)** UMAP presentation of three NK cells subtypes. **(B)** Feature plots showed the expression of canonically cell marker genes used to define each subtype. **(C)** Histogram showed the proportion of KLRF1+CD160 NK cells and TRGC1+TRDV2 NK cells in three groups. **(D)** Bubble plot shows the expression levels of cytotoxicity genes of NK cells in three groups. **(E)** Bubble plot shows the expression levels of complement receptor genes and immunoglobulin genes of NK cells in three groups. **(F)** GO enrichment analysis of NK cells between KPN_ACU and HC groups. **(G)** GO enrichment analysis of NK cells between KPN_ACU and KPN_REC groups. **(H)** Violin plots of IFNG genes in five type cells. **(I)** Violin plots of IFNG genes between KLRF1+CD160 NK cells and TRGC1+TRDV2 NK cells (up) and changes in three groups (down). **(J)** Violin plots of IFNGR1 and IFNGR2 genes in five type cells.

NK cells are important cytotoxic cells in peripheral blood, which can kill bacteria and bacteria infected cells (30). Our study found that the proportion of NK cells did not change significantly among HC, KPN_ACU and KPN_REC groups (**Figure 2C**). NK cells were divided in two biologically relevant subtypes: FCER1G+CD160 NK cells (FCER1G^+^CD160^+^), TRGC1+TRDV2 NK cells (TRGC1^+^TRDV2^+^) (**Figures 7A, B**). Although no significant differences were observed in the proportion of the NK subtypes between the different study groups, we observed that the proportion of FCER1G+CD160 NK cells tended to decrease during acute stage and partially recover during the recovery stage. Conversely, TRGC1+TRDV2 NK cells showed an increase during acute stage, and a decreasing trend during recovery stage (**Figure 7C**).

The function of the NK cells seems to change following disease progression. During acute stage, the expression of cytotoxicity- related genes decreased (**Figure 7D**), while the expression of complement receptor- and immunoglobulin- related genes was increased and their pathways upregulated (**Figures 7E, F, G**). This suggests that the killing ability of NK cells during acute stage may be reduced, and their role may be more focused on complement activation and immunoglobulin secretion. During recovery stage, the expression of complement receptor genes and immunoglobulin related genes in decreased to levels similar to the HC group (**Figure 7E**), indicating that the related function of complement and immunoglobulin in NK cells downregulated in the recovery stage of the disease. We detected the complement content among the three groups, and C1q, C3 and B factor were all decreased in KPN_ACU and KPN_REC, with statistical significance compared with HC (**Figure 6E**; **Supplementary Figure S4B**). It may be that with the development of the disease, a large amount of complement is consumed, which causes the compensatory expression of the complement receptor gene to increase. During the recovery stage, the complement content did not return to normal, and the complement receptor gene C3AR1, C5AR1 in KPN_REC group was significantly downregulated compared with KPN_ACU group (**Figures 6E**, **7E**; **Supplementary Figure S4B**).

In this study, the serum IFN-γ content in the KPN_ACU group was significantly higher than that in the HC group (**Figure 1I**). By analyzing the expression of the IFN-γ gene (IFNG), we found that IFNG was mainly expressed in NK cells during acute and recovery stages (**Figure 7H**), and that the expression levels of IFNG were similar between the two NK subtypes (**Figure 7I**). Furthermore, GO analysis found upregulation of a positive regulation of interferon-gamma production in NK cells (**Figure 7F**). Still, the IFN receptors IFNGR1 and IFNGR2 were not significantly upregulated in NK cells but significantly upregulated in monocytes during acute stage (**Figure 7J**), indicating that secreted IFN might have an effect on monocytes but not in NK cells. IFNG gene expression in NK cells during recovery stage was downregulated compared to the KPN_ACU group, but it was still higher than that in HC group (**Figures 7H, I**). IFN-γ content in serum was also decreased in KPN_REC group, which showed no statistical difference compared with HC group (**Figure 1H**), indicating that IFN-γ content in serum decreased faster than IFNG gene recovery. Some patients in the three groups had low serum levels of IFN-α (concentration less than 1pg/ml), while some patients had no serum levels of IFN-α (concentration 0pg/ml) (**Supplementary Table 5**). IFN-α is mainly produced by mononuclear-macrophages. Although the proportion of monocytes in patients with acute infection and recovery increased significantly, *K. pneumoniae*, as a Gram-negative bacterium, did not induce the production of a large amount of IFN-α in patients during the infection and recovery stage. Therefore, the immune defense function of *K. pneumoniae* bloodstream infection sepsis is mainly IFN-γ rather than IFN-α.

In conclusion, the cytotoxic capacity of NK cells is decreased in patients with bloodstream infection of *K. pneumoniae* with sepsis. NK cells are the main IFN-γ secreting cells in PBMCs, and IFN-γ, rather than IFN-α, plays an important defense role in *K. pneumoniae*.

## Discussion

Sepsis is a systemic severe inflammatory response syndrome with a mortality rate of 15-25%, and bloodstream infections are one of the most common causes of sepsis in humans (3, 5). *K. pneumoniae* is a Gram-negative bacterium and a major cause of sepsis through bloodstream infection (3). So far, the dynamic changes suffered by PBMCs during *K. pneumoniae* acute and recovery stages have not been investigated using scRNA-seq. In this study, we present a comprehensive and integrated single-cell landscape of peripheral immune responses to reveal changes in PBMCs composition and function during acute and recovery stages, enabling us to delve deeper into immune cell dysfunction and better understand the entire course of disease.

Our study found that *K. pneumoniae* bloodstream infections were predominant in the elderly, with an estimated mortality rate around 57%. CRP, PCT, D-Dimer, WBC, PMN% were significantly increased in patients with acute stage, indicating an obvious inflammatory response, while Lym% was decreased, suggesting immunosuppression of T lymphocytes. Although body temperature returned to normal during the recovery stage, the proportion of the different PBMC cell types and the expression of inflammatory makers did not reach normal healthy levels. These results suggest that patients may take longer to recover.

The analysis of the scRNA-Seq data revealed that during acute stage, T cells were depleted, with s obvious signs of exhaustion and apoptotic. This is consistent with previous studies that observed that a prolonged exposure to antigenic stimulation and pro-inflammatory cytokines leads to T cell exhaustion and apoptosis (18). CD4+ T cells can activate B cells to differentiate into plasma cells and memory B cells (31). Recent studies have found that the activity of CD4+ T cells in COVID-19 patients is normal (18), but in this study, the immune auxiliary function of CD4+ T cells was inhibited. This suggests that *K. pneumoniae* has different immune function effects on CD4+ T cells than COVID-19, which may lead to B cell dysfunction. MHC-II gene expression of T cells was significantly upregulated during acute stage, and subtype analysis showed that upregulation mainly occurred in CD8+ T cells rather than CD4+ T cells. The cytotoxic function of CD8+ T cells is upregulated, and our results also prove that CD8+ T cells are the main cells that play cytotoxic function in T cells. Upregulation of these genes and biological processes enables CD8+ T cells to recognize *K. pneumoniae* antigenic peptide and clear infected cells. Combining these results, we believed that CD4+T cells lead to T cell immunosuppression, while CD8+T cells lead to T cell activation. Therefore, this simultaneous phenomenon of immunosuppression and activation in T cells was caused by the dysfunction of different cell subtypes. We identified a new cytotoxic T cell subtype, STMN1+TUBA1B T cells, whose function needs further investigation. During the recovery stage, the proportion of T cells and their subtypes gradually recovered, but the apoptosis, depletion and cytotoxic genes were still at a high level, indicating that T cell function tended to be normal but had not recovered to the level of healthy people, and the recovery rate of T cell proportion was faster than that of gene recovery.

During the acute infection stage, the serum levels of IFN-γ and TNF-α were increased, and the receptor genes for these cytokines were found upregulated in monocytes. We believe these two concurrent events led to an enhanced interaction between IFN-γ, TNF-α and monocytes, resulting in an improved phagocytic ability of monocytes during sepsis (32), and the production of IL-1β. The production of these pro-inflammatory cytokines can promote the antigen presentation function of monocytes, a key mechanism in the resolution of an infection (23, 33). However, our gene expression results suggest that the antigen presentation ability of monocytes is decreased during acute sepsis. This may be inhibited by elevated IL-1RA in serum, which reduced the ability of the fungus to be cleared by the body (34), and our results suggest that elevated IL-1RA may also lead to reduced clearance of *K. pneumoniae*. A downregulation of the MHC-II gene in monocytes, as the one observed in our study, is an indicator of severe acute disease characterized by a “cytokine storm” (35). Decreased antigen presentation in monocytes also leads to T cell dysfunction, thereby inhibiting downstream adaptive immunity, which is detrimental to viral clearance and the relief of systemic inflammation (36). In addition, high level of TNF-α can lead to the release of a large amount of superoxide in the body (23), resulting in decreased SOD consumption, these were confirmed by the elevated TNF-α and decreased SOD in the acute stage of our study. These results suggest that a reduced ability of patients to remove damaging oxygen free radicals. Currently, it is believed that diffuse intravascular coagulation caused by Gram-negative bacteria is caused by bacterial endotoxin stimulating the body to produce excessive TNF-α (23). We found that PF4+PPBP+ monocytes participated in the biological process of platelet activation and coagulation. Monocytes infected by influenza virus or HIV could initiate the coagulation process, and Bunyavirus could also activate monocytes to induce abnormal coagulation (37). Therefore, it is possible that the combined action of PF4+PPBP+ monocyte and TNF-α caused the increase of D-dimer in patients. The increased proportion of FCGR3A+ monocytes with enhanced cytotoxicity may play a major role in the defense of *K. pneumoniae*. During sepsis recovery, the monocytes remained highly phagocytic and with an improved antigen presentation function. Additionally, serum IL-1β, IL-1RA, D-Dimer levels were significantly reduced, indicating that monocyte immunosuppression was reduced, and the pathways related to platelet activation and coagulation function were restored.

Serum levels of MCP-1 were elevated in patients with sepsis (38), and our results also showed this during the acute infection stage. MCP-1 was observed to act as chemotactic for DCs migration (38, 39). In our experiment the expression of MCP-1 receptor CCR2 in DCs was downregulated, explaining the decreased migration ability observed in the DCs. Furthermore, the reduced migration of DCs would limit their interaction with T cells in peripheral blood and subsequent activation of by antigen (37). In fact, we observed that during acute stage, the antigen presentation ability of DCs was downregulated, and the expression of exhaustion-related genes was upregulated, indicating that the DCs were immunosuppressed, and their capacity to efficiently recognize and present *K. pneumoniae* was impaired. The analysis of the DC subpopulations depicted not significant differences in the proportion of pDCs and cDCs between healthy individuals and patients undergoing sepsis or recovery. This was surprising because in a previous investigation, the peripheral blood of patients suffering tuberculosis, was depleted of pDCs (40). This difference in the innate immune response could be reflecting the differences in the bacterial cell wall, as *Mycobacterium tuberculosis* (causative agent of tuberculosis) is a Gram-positive bacterium. CCR2 gene encoding a chemokine receptor that binds, among others, to MCP-1 (25), was mainly expressed in pDCs, suggesting an important role of pDCs in antigen presentation during *K. pneumoniae* infection. In the recovery stage, the normal expression CCR2 gene of DCs was rapidly restored, the exhaustion genes were downregulated, and the expression of antigen presentation genes were higher than that in the other two groups of study. This indicates that the antigen presentation ability of DCs recovered faster than their depleted numbers. The recovery rate of cDCs antigen presentation genes was faster than that of pDCs, but the proportion of cells decreased, which may be caused by excessive consumption of cDCs. cDCs can cross-present antigens to CD8+ T cells, which may be the reason why CD8+ T cells maintain a high level of cytotoxic function during recovery stage. The role of cDCs in the defense of *K. pneumoniae* remains to be further studied.

B cells help clearing up bacterial infections by secreting antibodies and presenting antigens (28). During the acute *K. pneumoniae* infection stage, the expression of several exhaustion genes such as BCL2A1 in B cells was upregulated, indicating a potential cell exhaustion in this cell group. Furthermore, our study found that the expression of BCL2, a pro-survival factor of B cells (41), was decreased during acute stage, explaining the diminish proportion of B cells in that stage. CD4+ T cells can activate B cells to differentiate into plasma cells and memory B cells (20, 27). Hence, we hypothesized that B cells dysfunction during acute infection stage may be caused by the combination of B cell exhaustion and reduced helper function of CD4+ T cells. In turn, B cell exhaustion may reduce interferon secretion, complement and antibody immune response capacity (41). The expression of complement genes CR1 and C5AR1 genes in B cells was found downregulated during acute stage, and the concentration of C3 and other complement molecules in serum was also decreased. Complement depletion leads to a reduced complement mediated B cell activation and a defective immune regulation and cell differentiation (42). Interestingly, the antigen presentation capacity of the B cells was upregulated during acute stage, while that of monocytes and DCs was downregulated. This interesting result requires further study. In the recovery stage, B cells were still observed in low numbers, continue to suffer cell exhaustion, and with limited interferon secretion and complement responses. We identified a new type of CD2+IL32 B cells, which have obvious signs (BANK1, MEF2C) of activation during acute and recovery stages. Additionally, CD2+IL32 B cells maintain a high level of antigen presentation ability, suggesting a major role of *K. pneumoniae* during blood stream infections.

NK cells are important cytotoxic cells in the body (43). During *K. pneumoniae* acute infection stage, the granzyme-mediated cytotoxicity of NK cells is decreased, while immunoglobulin related genes were upregulated, suggesting that the antibody-dependent cell-mediated cytotoxicity (ADCC) of NK cells may be enhanced. The expression of the IFNG gene in both subtypes of NK cells was significantly upregulated, and the content of IFN-γ in serum was significantly increased, suggesting NK cells were the main producers of IFN-γ in PBMCs as previously described (43). The expression of monocyte IFN-γ receptor gene was upregulated during acute stage. This, together with the higher levels of IFN-γ found in serum, could explain the enhanced phagocytic activity of monocytes that we encountered in our study (32). Type I IFNs (mainly IFN-α) play an important role activating B and T cells in the clearance of COVID-19 and Bunyavirus (36, 37). In this study, Type I IFN was only detected in the serum of some patients during acute and recovery stages. So IFN-γ, rather than IFN-α, plays an important defense role in *K. pneumoniae*.

To finish, some of the limitations of our investigation are that this is a single center study with a limited number of patients with *K. pneumoniae* bloodstream infection. Moreover, these patients have very serious conditions, which makes the follow up difficult therefore decreasing the number of cases for the study. Our newly discovered subtypes of B and T cells have not been further studied and validated. Three of the seven patients in the KPN_ACU group eventually survived. According to some reports in the literature, there are some differences in the transcriptome of immune cells between surviving and dead patients infected with the same pathogen. The prognosis of patients can be predicted based on DEGs and some cellular markers. The difference in immune status of PBMCs between surviving and deceased patients infected with *K. pneumoniae* is also the focus of our follow-up studies. This will be the topic of future work.

In summary, we conducted a systematic study on the dynamic changes in immune function of PMBCs from patients suffering sepsis caused by *K. pneumoniae* bloodstream infection using scRNA-seq for the first time. In the acute infection and recovery stages, we found that the immune function of CD4+ T cells and the cytotoxic function of NK cells mediated by granzyme were inhibited, while CD8+ T cells were mainly cytotoxic cells. The antigen presentation function of monocytes and DCs was inhibited, leaving B cells as the main antigen presentation among the PBMCs. The phagocytosis function of monocytes was enhanced however. IFN-γ, rather than IFN-α, plays an important defense role in *K. pneumoniae*. Overall, our results indicated that there are both pro-inflammatory and immunosuppressive conditions in the acute infection stage, and the gene and cell levels of patients still did not return to normal state in the recovery stage. This provides the immunological basis for the treatment and rehabilitation of *K. pneumoniae*.

## Data availability statement

The data that support the findings of this study have been deposited into CNGB Sequence Archive (CNSA) of China National GeneBank DataBase (CNGBdb) with accession number CNP0005648.

## Ethics statement

The studies involving humans were approved by Ethics Committee of Dongfang Hospital of Beijing University of Chinese Medicine. The studies were conducted in accordance with the local legislation and institutional requirements. The participants provided their written informed consent to participate in this study.

## Author contributions

SGZ: Data curation, Formal analysis, Investigation, Resources, Writing – original draft, Writing – review & editing. NZ: Visualization, Writing – review & editing, Conceptualization, Investigation, Resources. JH: Resources, Writing – review & editing. ZS: Methodology, Writing – review & editing, Software. HJ: Methodology, Writing – review & editing, Software. WH: Resources, Writing – review & editing. DK: Resources, Writing – review & editing, Methodology. QL: Methodology, Writing – review & editing, Resources. YR: Writing – review & editing, Resources. SHZ: Writing – review & editing, Formal analysis, Software, Conceptualization. YJ: Funding acquisition, Supervision, Writing – review & editing, Conceptualization. PL: Funding acquisition, Supervision, Writing – review & editing, Conceptualization, Resources.

## Supplementary material

Supplementary Figure S1 Gene expression level in T cells and subtypes. **(A)** Bubble plot shows the expression levels of antigen presentation genes of T cells in three groups. **(B)** Bubble plot shows the expression levels of antigen presentation genes of CD8+ T cells in three groups. **(C)** Bubble plot shows the expression levels of antigen presentation genes of CD4+ T cells in three groups. **(D)** Bubble plot shows the expression levels of cytotoxicity genes of STMN1+TUBA1B T cells in three groups.

Supplementary Figure S2 Biological process and gene expression of monocytes and subtypes. **(A)** GO enrichment analysis of monocytes between KPN_ACU and KPN_REC groups. **(B)** GO enrichment analysis between KPN_REC and HC groups. **(C)** Violin plots of IL1B and IL18 genes in five cells. **(D)** Violin plots of IL1RN and IL18 genes in five cells between three groups. **(E)** Violin plots of IL1RN genes in four subtypes of monocytes among three groups. **(F)** Bubble plot shows the expression levels of antigen presentation genes of monocytes in three groups. **(G)** Heatmap showed the maker genes in four subtypes. **(H)** Violin plots of TNFRSF1B genes in five cells between three groups.

Supplementary Figure S3 Changes in transcriptional function and proportion of cDCs and pDCs in three groups. **(A)** Violin plots of canonical annotation marker genes (columns) for different subtypes (rows). **(B)** Proportions of cDCs and pDCs in three groups. **(C)** Bubble plot shows the expression levels of antigen presentation genes of pDCs in three groups. **(D)** Bubble plot shows the expression levels of antigen presentation genes of cDCs in three groups. **(E)** Violin plots of CCR2 genes of cDCs and pDCs in three groups.

Supplementary Figure S4 The changes of transcriptome and cell proportion and interaction with complement in B cells and their subtypes. **(A)** Violin plots of IFN genes of B cells in three groups. **(B)** The level of B Factor (left) and C1q (right) in serum of three groups. **(C)** Violin plots of canonical annotation marker genes (columns) for B cells subtypes (rows). **(D)** Violin plots shows antigen presentation module score in three groups. **(E)** Proportions of the three B cells subtypes. **(F)** GO enrichment analysis of CD2+IL32 B cells between KPN_ACU and HC groups. **(G)** GO enrichment analysis of CD2+IL32 B cells between KPN_REC and HC groups.
