# Supplementary figures and images for: Dynamic immune status analysis of peripheral blood mononuclear cells in patients with Klebsiella pneumoniae bloodstream infection sepsis using single-cell RNA sequencing
Source: Front Immunol. 2024 Jun 5;15:1380211. doi: 10.3389/fimmu.2024.1380211 (PMC11185935; doi:10.3389/fimmu.2024.1380211)

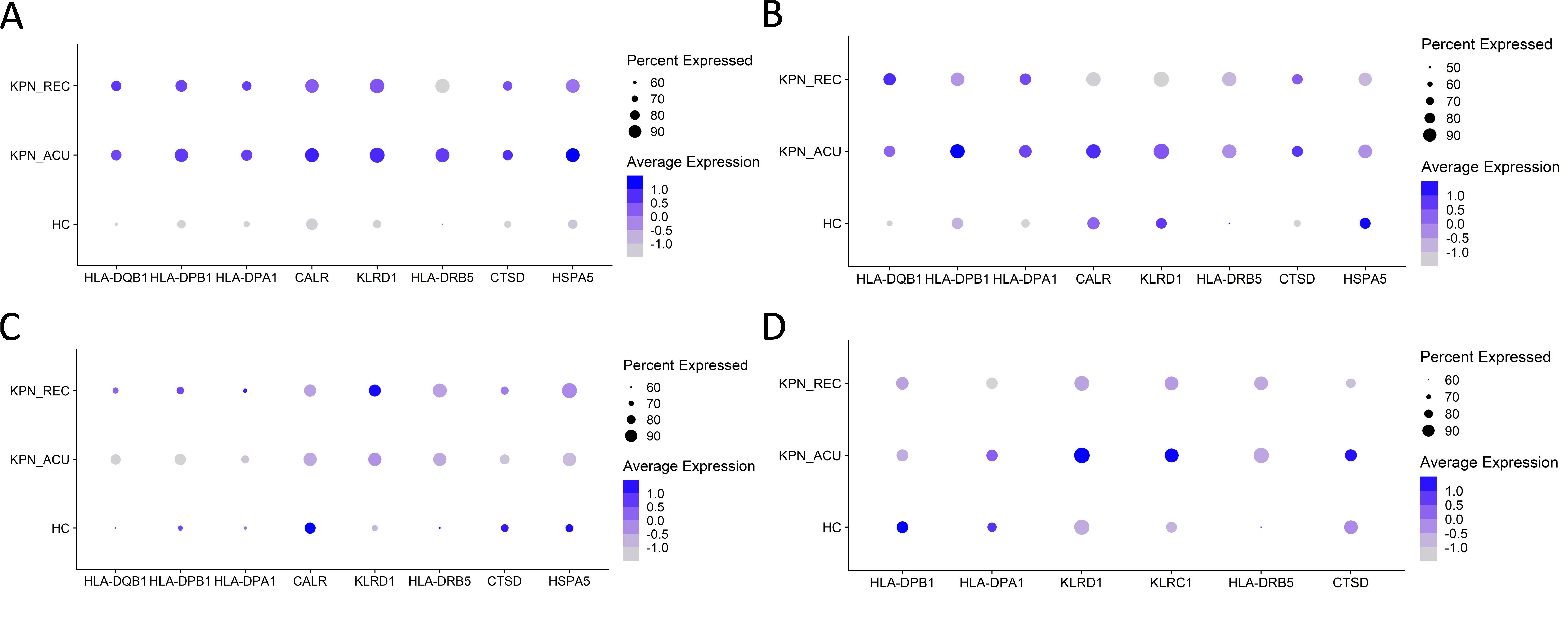

Supplement: Supplementary Figure S1 — Gene expression level in T cells and subtypes. (A) Bubble plot shows the expression levels of antigen presentation genes of T cells in three groups. (B) Bubble plot shows the expression levels of antigen presentation genes of CD8+ T cells in three groups. (C) Bubble plot shows the expression levels of antigen presentation genes of CD4+ T cells in three groups. (D) Bubble plot shows the expression levels of cytotoxicity genes of STMN1+TUBA1B T cells in three groups. [file Image_1.jpeg]

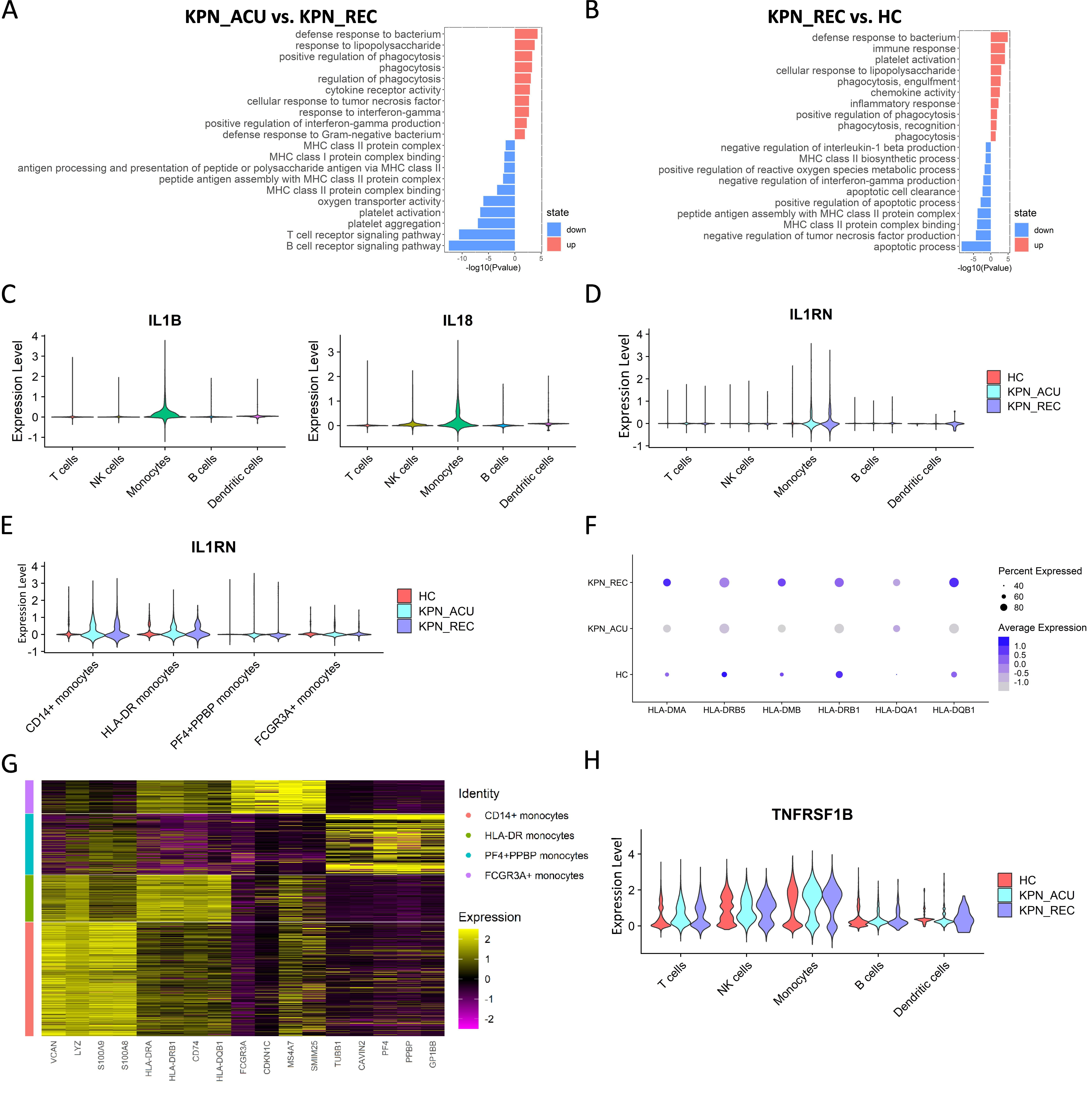

Supplement: Supplementary Figure S2 — Biological process and gene expression of monocytes and subtypes. (A) GO enrichment analysis of monocytes between KPN_ACU and KPN_REC groups. (B) GO enrichment analysis between KPN_REC and HC groups. (C) Violin plots of IL1B and IL18 genes in five cells. (D) Violin plots of IL1RN and IL18 genes in five cells between three groups. (E) Violin plots of IL1RN genes in four subtypes of monocytes among three groups. (F) Bubble plot shows the expression levels of antigen presentation genes of monocytes in three groups. (G) Heatmap showed the maker genes in four subtypes. (H) Violin plots of TNFRSF1B genes in five cells between three groups. [file Image_2.jpeg]

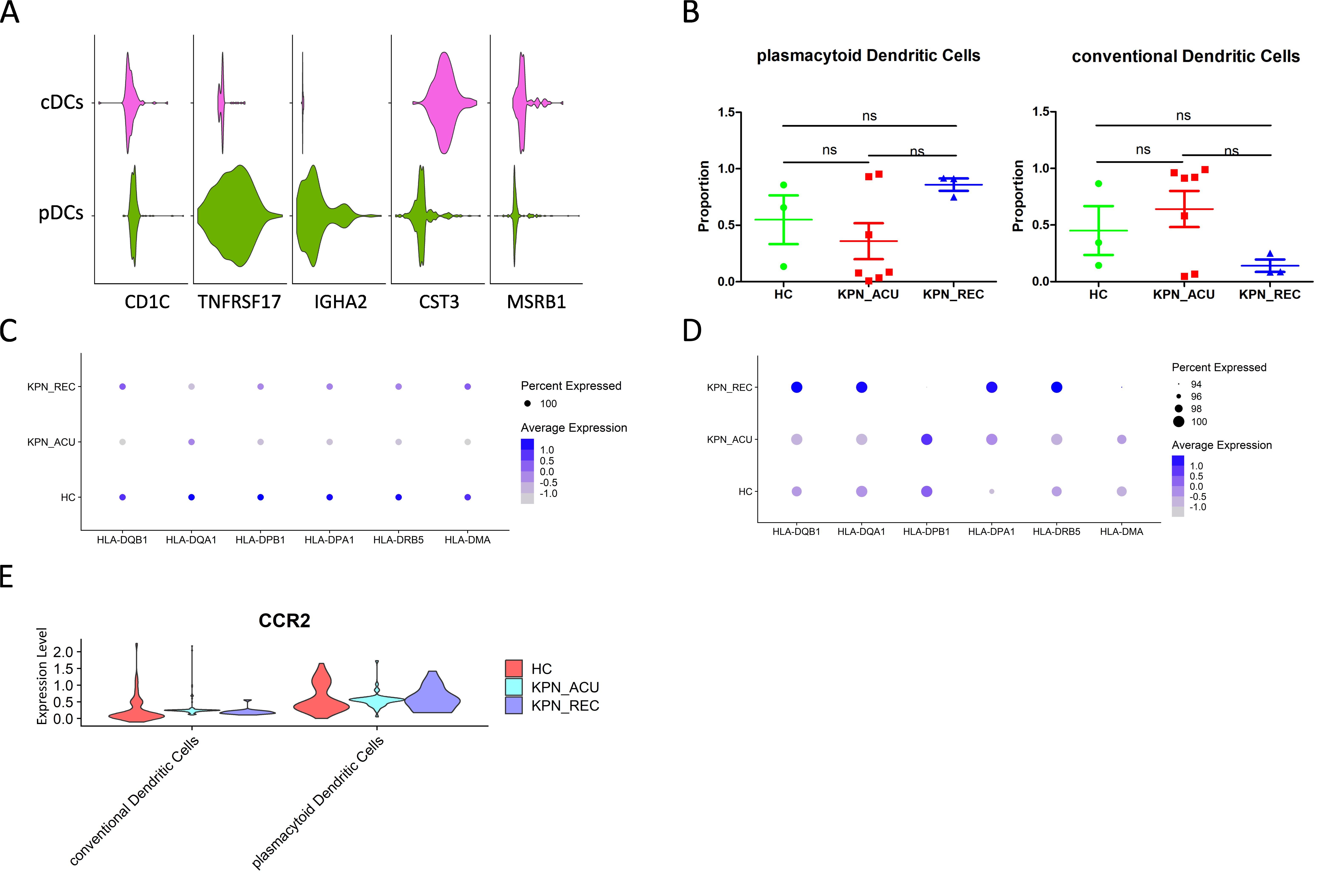

Supplement: Supplementary Figure S3 — Changes in transcriptional function and proportion of cDCs and pDCs in three groups. (A) Violin plots of canonical annotation marker genes (columns) for different subtypes (rows). (B) Proportions of cDCs and pDCs in three groups. (C) Bubble plot shows the expression levels of antigen presentation genes of pDCs in three groups. (D) Bubble plot shows the expression levels of antigen presentation genes of cDCs in three groups. (E) Violin plots of CCR2 genes of cDCs and pDCs in three groups. [file Image_3.jpeg]

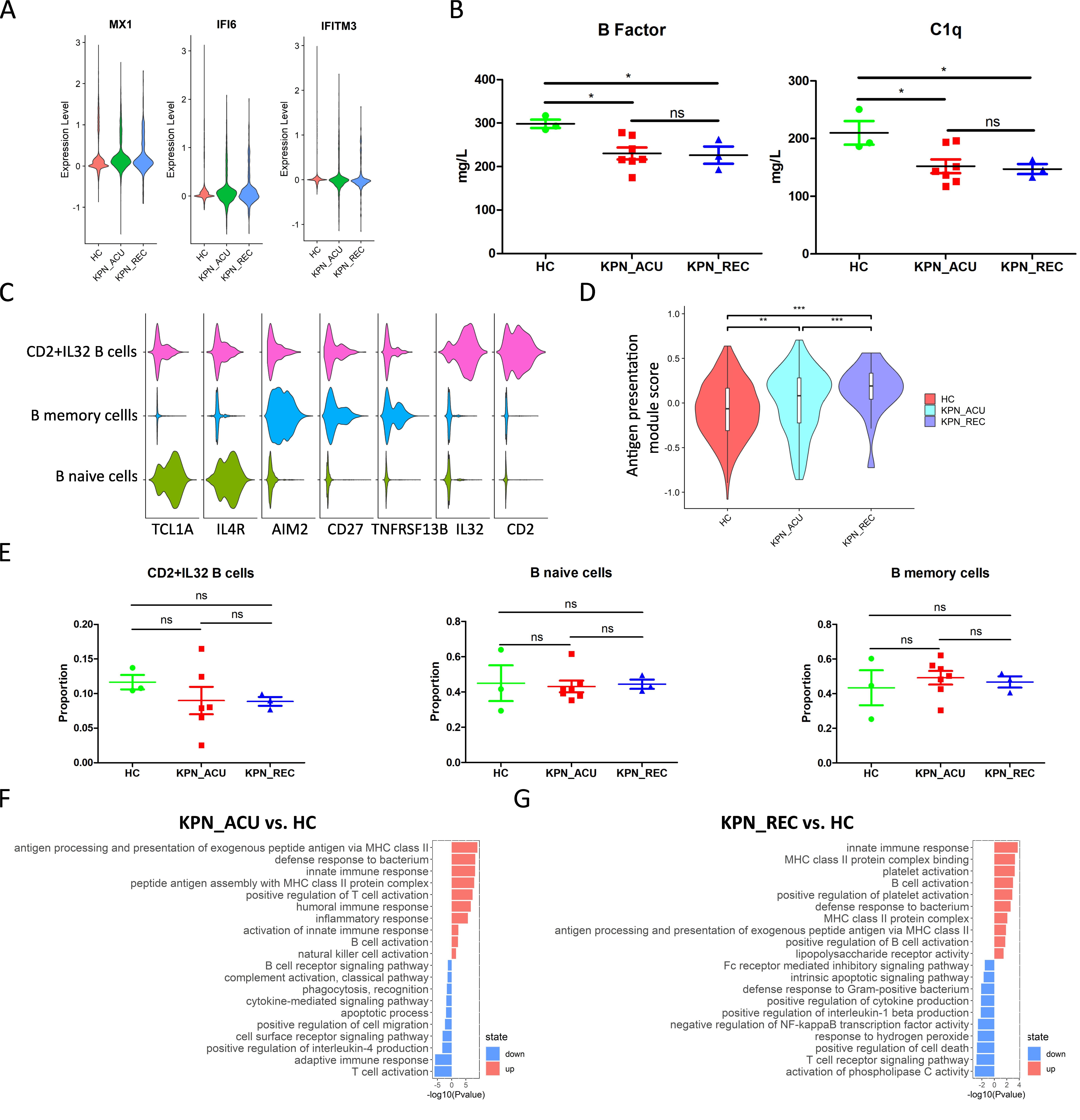

Supplement: Supplementary Figure S4 — The changes of transcriptome and cell proportion and interaction with complement in B cells and their subtypes. (A) Violin plots of IFN genes of B cells in three groups. (B) The level of B Factor (left) and C1q (right) in serum of three groups. (C) Violin plots of canonical annotation marker genes (columns) for B cells subtypes (rows). (D) Violin plots shows antigen presentation module score in three groups. (E) Proportions of the three B cells subtypes. (F) GO enrichment analysis of CD2+IL32 B cells between KPN_ACU and HC groups. (G) GO enrichment analysis of CD2+IL32 B cells between KPN_REC and HC groups. [file Image_4.jpeg]
